# Acute B Lymphoblastic Leukemia Developing in Patients with Multiple Myeloma: Presentation of Two Cases

**DOI:** 10.4274/tjh.galenos.2019.2019.0018

**Published:** 2019-11-18

**Authors:** Jiang Mei, Li Na, Ji Dexiang, Li Fei, Zhang Zhanglin

**Affiliations:** 1 The First Affiliated Hospital of Nanchang University, Department of Clinical Laboratory, Nanchang, P.R. China; 2 The First Affiliated Hospital of Nanchang University, Department of Stomatology, Nanchang, P.R. China; 3 The First Affiliated Hospital of Nanchang University, Department of Hematology, Nanchang, P.R. China; ✩ Jiang Mei and Li Na contributed equally to this study.

**Keywords:** Acute lymphoblastic leukemia, Multiple myeloma, Therapy-related, Genetics, Immunophenotyping

## To the Editor,

Therapy-related acute myeloid leukemias (t-AMLs) following therapy are well described in the literature, but only rare cases of therapy-related acute lymphoblastic leukemia (t-ALL) have been reported previously. Cases of multiple myeloma (MM) terminating in ALL are even rarer. Herein, we report the clinicopathological, immunological, cytogenetic, and molecular features of two patients diagnosed with B-cell acute lymphoblastic leukemia (B-ALL) and MM who presented with MM at the initial diagnosis.

Patient 1, a 68-year-old male, was diagnosed with MM in 2015. He received 2 cycles of PD (bortezomib and dexamethasone) with a good response, and then maintenance with thalidomide.

Patient 2, a 65-year-old female, was diagnosed with MM in 2012. She received 4 cycles of VAD (vincristine, epirubicin, and dexamethasone) with a partial response. She then relapsed and received treatment with one cycle of TAD (thalidomide, epirubicin, and dexamethasone). After that, she achieved complete remission. In 2016, the patient relapsed again. She continued treatment with BTD (bortezomib, dexamethasone, and thalidomide) and achieved partial response.

**Figure 1:**
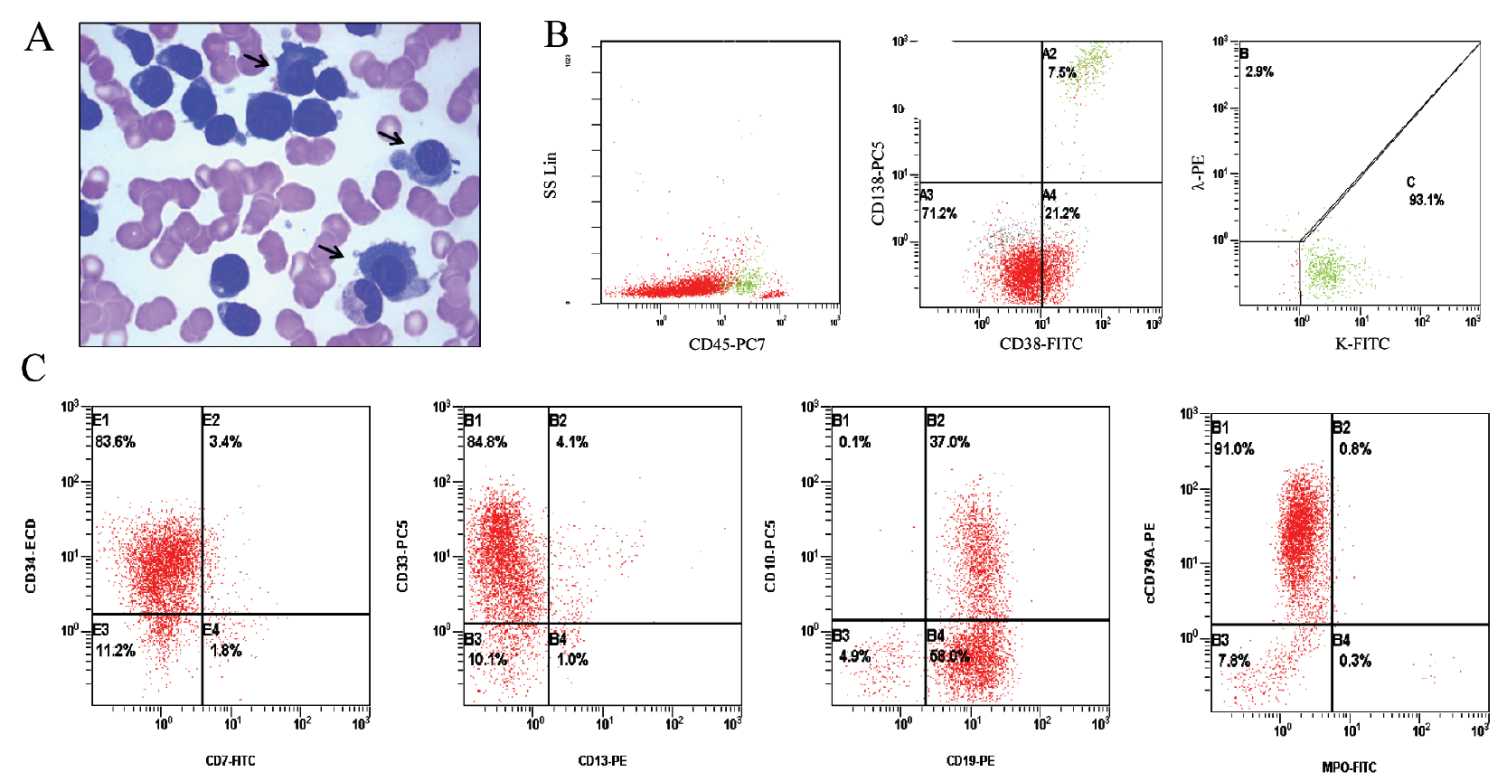
Patient 1: A) Black arrows point at malignant plasma cells, which are very different from other lymphoblasts (Wright-Giemsa staining, 100x). B) Malignant plasma cells were positive for CD38, CD138, and monoclonal kappa (green region of the scatter plot). C) The lymphoblasts were immunophenotyped as B-cell and expressed CD10, CD19, CD34, and cCD79a with aberrant coexpression of CD33. The morphologic and immunological characteristics of Patient 2 were similar.

In 2017, the two patients both presented with leukopenia. Immunofixation electrophoresis showed monoclonal IgG and K light chain. The bone marrow was heavily infiltrated by lymphoblasts and a few malignant plasma cells. Flow cytometry analysis demonstrated that malignant plasma cells with CD38, CD138, and monoclonal K chain and B-cell lymphoblasts expressed CD10, CD19, CD34, HLA-DR, cCD79a, and CD33. No other aberrant expression of myeloid or T lymphocyte-associated antigens was identified (Figures 1A-1C). The female patient’s G-banding cytogenetic results revealed a hypodiploid and complex karyotype. Reverse transcription-polymerase chain reaction for detection of fusion genes in the two patients was negative. Both patients were diagnosed with B-ALL with MM. The male patient declined any treatment due to poor performance status and died four months later. The female patient was treated with low-dose chemotherapy (vincristine, epirubicin, dexamethasone, and bortezomib) and did not respond well; she died one month later. The clinical features of the two patients are summarized in Table 1.

It has been reported in the literature that therapy-related acute leukemia comprises 2 major types: alkylating agent/radiotherapy-related and topoisomerase II inhibitor-related [1]. Alkylating agent-related acute leukemia is associated with abnormalities of chromosomes 5 and/or 7, while topoisomerase II inhibitor-related acute leukemia has been linked to 11q23 [2,3]. The female patient received a topoisomerase II inhibitor while the male did not, and neither of them showed specific genetic abnormalities. Intriguingly, the clinical, morphologic, and immunological characteristics of the two patients were similar. MM is a plasma cell neoplasm derived from mature B-lymphocytes, whereas B-ALL is a B-cell neoplasm derived from early B-precursors. It is possible that MM and B-ALL may derive from the same stem cell clones, or MM cells dedifferentiate into immature B cells that develop B-ALL [4]. They may have identical karyotypes and immunophenotyping, and they may share some cytogenetic abnormalities [5,6]. The possibility of MM and B-ALL deriving from two independent clones cannot be excluded, either. Future studies such as molecular and cytogenetic studies to explore their relationship would be intriguing.

## Figures and Tables

**Table 1:**
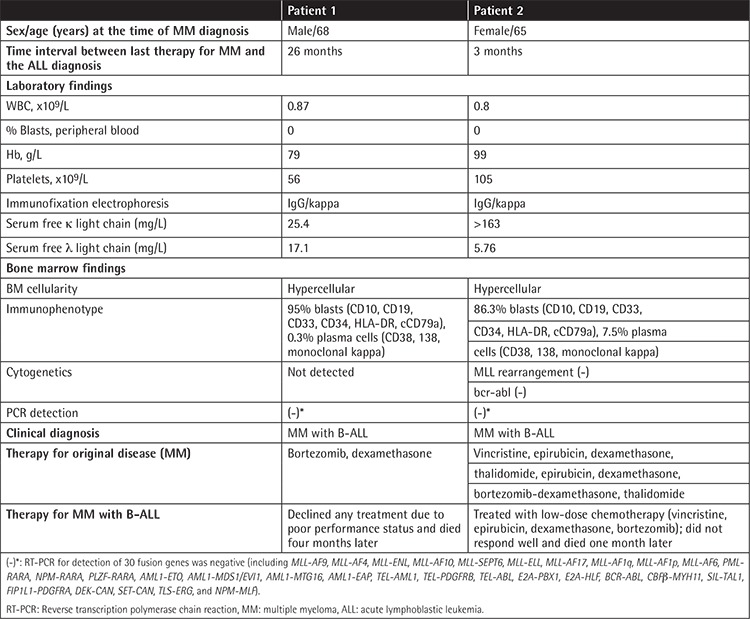
Clinical features of acute lymphoblastic leukemia in patients with previously treated multiple myeloma (MM) from our institution.
